# Podocalyxin-Like Protein 1 Regulates TAZ Signaling and Stemness Properties in Colon Cancer

**DOI:** 10.3390/ijms18102047

**Published:** 2017-09-23

**Authors:** Wen-Ying Lee, Chih-Chia Kuo, Bo-Xing Lin, Chia-Hsiung Cheng, Ku-Chung Chen, Cheng-Wei Lin

**Affiliations:** 1 Department of Cytopathology, Chi Mei Medical Center, Tainan 710, Taiwan; d940232@mail.chimei.org.tw; 2 Department of Pathology, School of Medicine, College of Medicine, Taipei Medical University, Taipei 110, Taiwan; 3 Department of Biochemistry and Molecular Cell Biology, School of Medicine, College of Medicine, Taipei Medical University, Taipei 110, Taiwan; b507101052@tmu.edu.tw (C.-C.K.); xzx22668844@gmail.com (B.-X.L.); chcheng@tmu.edu.tw (C.-H.C.); kuchung@tmu.edu.tw (K.-C.C.); 4 Graduate Institute of Medical Sciences, College of Medicine, Taipei Medical University, Taipei 110, Taiwan; 5 Center for Cell Therapy and Regeneration Medicine, Taipei Medical University, Taipei 110, Taiwan

**Keywords:** colon cancer, epithelial-mesenchymal transition, podocalyxin, TAZ, cancer stem cells

## Abstract

Colon cancer is the third most common cancer in the world and the second most common cause of cancer-related mortality. Molecular biomarkers for colon cancer have undergone vigorous discovery and validation. Recent studies reported that overexpression of podocalyxin-like protein 1 (PODXL) is associated with distant metastasis and poor prognosis across several types of malignancies. Its role and underlying molecular mechanism, however, are not yet fully understood. In the present study, we revealed that the Hippo transducer, the transcriptional coactivator with PDZ-binding motif (TAZ), acts as a downstream mediator of PODXL in colon cancer. Inhibition of PODXL resulted in the suppression of TAZ signaling and the downregulation of Hippo downstream genes. Moreover, PODXL plays a critical role in cancer stemness, invasiveness, and sensitivity to chemotherapies in colon cancer HCT15 cells. Notably, expression of PODXL showed a positive correlation with stem-like and epithelial-mesenchymal transition (EMT) core signatures, and was associated with poor survival outcomes in patients with colon cancer. These findings provide novel insights into the molecular mechanism of PODXL-mediated tumorigenesis in colon cancer.

## 1. Introduction

Colon cancer is the third most common cancer in the world and the second most common cause of cancer-related mortality. Genetic mutations in tumor suppressor genes such as *adenomatous polyposis coli* (APC) and *TP53*, and oncogenic mutations in *KRAS*, *BRAF*, or *PI3KCA,* are identified to be crucial for the development and progression of colon cancer [1,2]. Despite the improvement of surgical or drug treatments in colon cancer, the overall survival is still limited. Therefore, it is an urgent demand to identify novel biomarkers as diagnostic methods and a therapeutic target for colon cancer.

Podocalyxin-like protein 1 (PODXL), a cell surface glycoprotein belonging to the CD34 family, was first identified in kidney glomerular epithelial cells. As to the cellular function of PODXL, it dominantly participates in regulating cell mobility through orchestrating the actin polymerization complex including ezrin and the Na^+^/H^+^ exchanger regulatory factor (NHERF) [3,4]. Expression of PODXL is colocalized with submembrane actin in lamellipodia ruffles, or with vinculin at cell protrusions [5]. Conversely, the dissociation of PODXL from actin results in the loss of the glomerular foot formation and podocyte integrity [6]. Dysregulation of PODXL in tumor cells was reported to increase their aggressive and metastatic capabilities, and its overexpression is associated with poor survival in breast, colon, brain, and pancreatic cancers [7,8,9,10]. Although an association of PODXL having prognostic value in tumors was reported, the detailed underlying mechanism remains to be elucidated.

Studies reported that the Rho GTPase family plays an important role in the transduction of the downstream signaling of PODXL. We previously identified that PODXL regulates the activity of invadopodia in the breast tumor cell through Rac1/Cdc42/cortactin signaling [11]. Additionally, PODXL enhances the metastatic potential of renal cell carcinoma by recruiting the Rac1 guanine nucleotide exchange factor, ARHGEF7 [12]. Rho GTPase participates in the maturation of actin polymerization, which further transduces downstream signaling events, for example, Hippo signaling. The core Hippo pathway is composed by mammalian STE20-like protein kinase 1 (MST1), large tumor suppressor homologue 1 (LATS1), and two key effectors, viz., the yes-associated protein (YAP) and transcriptional coactivator with PDZ-binding motif (TAZ). The Hippo pathway becomes activated when MST1 phosphorylates LATS1, which then phosphorylates and induces proteasomal degradation of YAP/TAZ [13]. YAP and TAZ take center stage in regulating tumor growth, motility, self-renewal, and drug resistance [14,15]. Deregulation of the Hippo mechanism was demonstrated to be involved in several malignancies. Previous studies reported that actin cytoskeleton or cellular tension plays a crucial role in converging on the upstream signaling to the core Hippo cascade. Changes in cytoskeleton by mechanical cues were shown to regulate YAP/TAZ activity [16,17]. The activity of YAP is also reported to be regulated by a particular F-actin structure [18]. Because we have identified that PODXL activates Rac1/cdc42 and enhances actin polymerization [11], we hypothesized that the Hippo signaling may act as downstream signaling of PODXL. However, the association of PODXL with the Hippo signaling has not yet been identified.

In this study, we revealed that TAZ is a novel downstream signaling transducer of PODXL in mediating cancer invasiveness and stemness properties in colon cancer. Inhibition of PODXL suppresses TAZ and downstream genes expression. Therapy which targets either PODXL or TAZ may have the potential to treat colon cancer.

## 2. Results

### 2.1. Expression of Podocalyxin-Like Protein 1 (PODXL) Is Associated with Transcriptional Coactivator with PDZ-Binding Motif (TAZ) Downstream Signaling and Poor Survival in Colon Cancer

**Figure 1:**
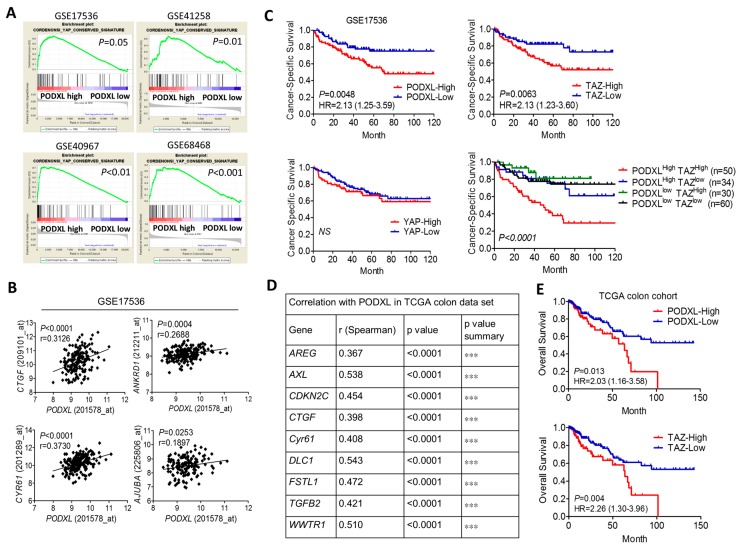
Expression of Podocalyxin-like Protein 1 (PODXL) is correlated with the Yes-associated Protein (YAP) signature and poor survival in colorectal cancer. (**A**) The gene set enrichment algorithm (GSEA) shows the association of PODXL with the -YAP signature in colorectal cancer (CRC). (**B**) Expression of PODXL was correlated with YAP/TAZ downstream genes in CRC. Data were retrieved from Gene Expression Omnibus (GEO) accession no. GSE17536. The correlation coefficient was determined by Spearman’s test. (**C**) Coexpression of PODXL and TAZ conferred poorer survival in CRC patients. Kaplan-Meier analysis of the cancer-specific survival of CRC patients according to PODXL and TAZ. The statistical difference was based on the log-rank test. (**D** and **E**) Expression of PODXL was correlated with TAZ downstream signaling (**D**) and poor overall survival outcomes (**E**) in the cancer genome atlas website (TCGA) colorectal cancer dataset (COADREAD). CTGF, connective tissue growth factor; CYR61, cysteine rich angiogenic inducer 61; ANKRD1, ankyrin repeat domain 1.

To examine whether the Hippo pathway is downstream of PODXL, we analyzed the correlation of PODXL with the YAP-conserved gene signature using a gene set enrichment algorithm (GSEA). Results showed that expression of PODXL was significantly associated with the YAP signature in four public colon cancer datasets (Figure 1A). The downstream target of Hippo signaling, including connective tissue growth factor (CTGF), cysteine rich angiogenic inducer 61(CYR61), ankyrin repeat domain 1 (ANKRD1), and AJUBA, was confirmed to be positively correlated with the PODXL expression level (Figure 1B). Moreover, colon cancer patients overexpressing PODXL were confirmed to be associated with poor survival outcomes (Figure 1C). Additionally, the survival probability was associated with TAZ, but not with YAP (Figure 1C). Furthermore, coexpression of PODXL and TAZ in colon cancer patients rendered the worst prognosis (Figure 1C). Similar results were obtained in the cancer genome atlas website (TCGA) colon cancer dataset (Figure 1D,E). These data suggest that PODXL might associate with Hippo signaling.

### 2.2. PODXL Regulates TAZ and Downstream Gene Expressions

**Figure 2:**
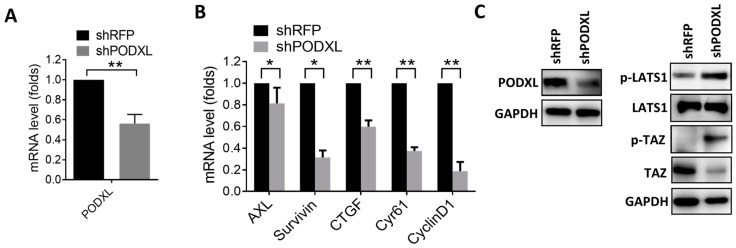
Suppression of PODXL decreases TAZ downstream gene expressions. (**A**) Real-time PCR analysis of the PODXL mRNA level in PODXL-knockdown HCT15 cells. (**B**) Knockdown of PODXL-downregulated TAZ downstream gene expressions, as measured by real-time PCR assay. * *p* < 0.05; ** *p* < 0.01, as assessed by an unpaired *t*-test. (**C**) Western blot analysis of protein levels of the Hippo cascade in PODXL knockdown HCT15 cells. GAPDH, glyceraldehyde 3 phosphate dehydrogenase; RFP, Red Fluorescent Protein.

To validate the correlation between PODXL and TAZ, expression of PODXL was silenced in HCT15 cells (Figure 2A). Results of the real-time PCR showed that inhibition of PODXL-downregulated TAZ downstream targets including *AXL, CTGF, CYR61, Survivin,* and *CyclinD1* (Figure 2B), confirming the association between PODXL and the TAZ signature in colon cancer patients. To identify the effect of PODXL on the Hippo signaling, we analyzed the level of TAZ and LATS1. Results showed that the phosphorylation of LATS1 and TAZ was increased in PODXL-knockdown cells, while total TAZ protein levels were reduced (Figure 2C), indicating that inhibition of PODXL might activate the Hippo mechanism.

### 2.3. Expression of PODXL Is Associated with the Stem Cell Signature in Colon Cancer

**Figure 3:**
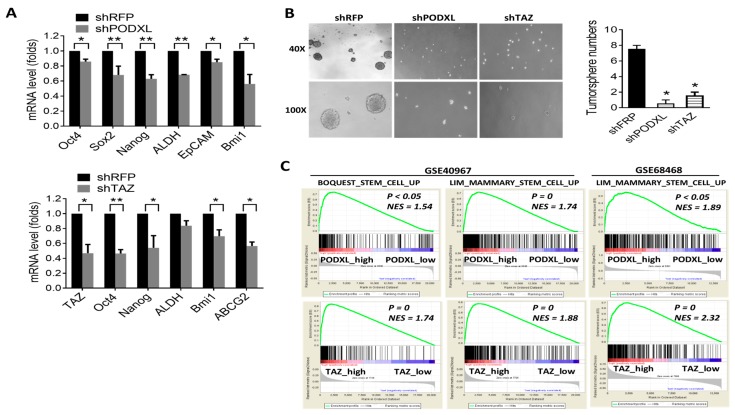
Expressions of PODXL and TAZ are associated with the stem cell signature in colorectal cancer. (**A**) Real-time PCR analysis of pluripotent stem cell markers and cancer stem-like gene expressions in PODXL- (upper panel) and TAZ-knockdown (lower panel) HCT15 cells. * *p* < 0.05; ** *p* < 0.01, as assessed by an unpaired *t*-test. (**B**) Knockdown of PODXL and TAZ-suppressed tumorsphere formation in HCT15 cells. Pictures were taken under a reverse microscope at 40× (upper panel) or 100× (lower panel) magnification. * *p* < 0.05 as assessed by an unpaired *t*-test. (**C**) Correlations of PODXL and TAZ with stem cell-related gene signatures. Data were retrieved from gene expression omnibus (GSE68468 and GSE40967). NES, normalized enrichment score.

Cancer stem cell play a crucial role in malignant progression of tumor cells. It was reported that overexpression of TAZ promotes cancer stemness property, and our data showed that PODXL regulates TAZ signaling. We next examined the effect of PODXL on stemness characteristics. Results showed that inhibition of PODXL-downregulated genes which associated with pluripotent stemness (*Oct4*, *Nanog*, and *Sox2*) and drug resistance (*ALDH*) (Figure 3A). Importantly, inhibition of PODXL significantly reduced the tumorsphere-forming capacity in HCT15 cells (Figure 3B), indicating that expression of PODXL is crucial for the self-renewal property of colon cancer cells. Consistently, inhibition of TAZ resulted in downregulation of stem-like gene expressions and tumorsphere formation, similar with that in PODXL knockdown cells (Figure 3A,B). Moreover, the GSEA revealed that expressions of PODXL and TAZ were significantly associated with the stem cell gene signature in colon cancer patients (Figure 3C).

### 2.4. PODXL Regulates Epithelial-Mesenchymal Transition (EMT) Gene Expressions and Aggressiveness in Colon Cancer

**Figure 4:**
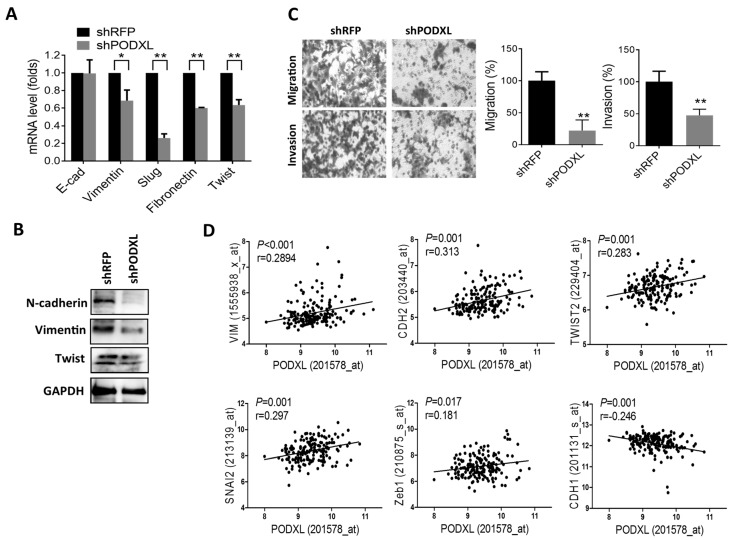
PODXL regulates epithelial-mesenchymal transition (EMT) gene expressions and tumor invasiveness. (**A** and **B**) Knockdown of PODXL-downregulated EMT-related gene expressions, as determined by real-time PCR (**A**) and Western blot (**B**) analyses. * *p* < 0.05; ** *p* < 0.01, as assessed by an unpaired *t*-test. (**C**) Inhibition of PODXL-suppressed tumor migration and invasion. Pictures were taken at 200x magnification. Data were derived from three random fields by three independent experiments, and results are expressed as multiples of the control. ** *p* < 0.01, as assessed by an unpaired *t*-test. (**D**) Scatter plot analyses of the correlation between PODXL and EMT-related gene expressions in colon cancer patients (GSE17536). The correlation coefficient was determined by Spearman’s test.

Given that induction of the EMT causes cells to have stem-like characteristics, we next examined the effect of PODXL on EMT gene regulation. Results showed that suppression of PODXL substantially inhibited EMT-related gene expressions (Figure 4A,B). Additionally, the migratory and invasive capabilities significantly decreased in PODXL-knockdown cells (Figure 4C). Moreover, we analyzed mRNA expression levels in patients with colon cancer, and results showed that expression of PODXL was positively correlated with mesenchymal markers such as vimentin (VIM), N-cadherin (CDH2), Twist2, Slug (SNAI2), and Zeb1, whereas it was negatively correlated with epithelial marker E-cadherin (CDH1) expressions (Figure 4D). These data indicate that PODXL regulates EMT gene expressions and the aggressiveness of colon cancer.

### 2.5. PODXL Confers Resistance to Conventional Chemotherapies

**Figure 5:**
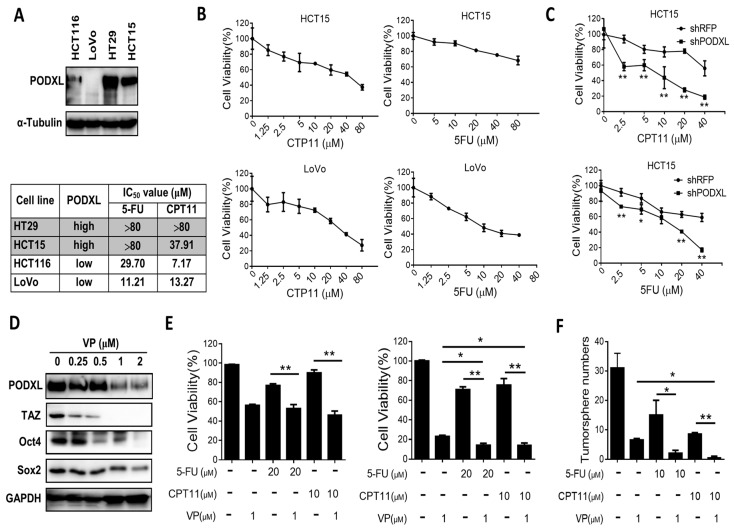
Expression of PODXL confers resistance to conventional chemotherapies in colorectal cancer. (**A**) Western blot analysis of the PODXL protein level in CRC cell lines. (**B**) Determination of 50% inhibitory concentration (IC_50_) values of 5-fluorouracil (5-FU) and irinotecan (CPT11) toward CRC cells. CRC cells were treated with various concentrations of 5-FU or CPT11 for 48 h, and cell viability was measured by MTT assay. Results are shown as the mean ± standard error of the mean (*n* = 3). The IC_50_ value was obtained using the GraphPad Prism5 program. (**C**) Knockdown of PODXL increased sensitivity in response to chemotherapies. 5-FU and CPT11 (0–40 μM) were added in mock and PODXL knockdown HCT15 cells for 48 h, and cell viability was measured by MTT assay. (**D**) HCT15 cells were treated with verteporfin (VP) for 24 h, and protein expression was analyzed by Western blotting. (**E**) HCT15 cells were treated with 5-FU or CPT11 in the presence or absence of VP for 24 (left panel) or 48 h (right panel), and cell viability was measured by MTT assay. Results are shown as the mean ± standard error of the mean (*n* = 3). (**F**) HCT15 cells were treated with 5-FU or CPT11 combined with VP, and formation of tumorsphere was assessed. A two-tailed Student’s *t*-test was applied to determine the statistical significance of the indicated groups. * *p* < 0.05, ** *p* < 0.01.

To further gain insight into the role of PODXL in cancer stemness characteristics, we evaluated the effect of PODXL on drug resistance, a hallmark of cancer stemness. Interestingly, we found HT29 and HCT15 colon cancer cells that expressed high level of PODXL were more resistant to 5-flurouracil (5-FU) and irinotecan (CPT11), two conventional chemotherapies widely used for colon cancer treatment (Figure 5A,B). On the contrary, HCT116 and LoVo cells, which expressed lower levels of PODXL, were more sensitive to 5-FU and CPT11 (Figure 5A,B). Importantly, suppression of PODXL substantially increased the sensitivity to chemotherapies (Figure 5C). To further validate the feasibility of inhibiting PODXL or TAZ for colon cancer treatment, we examined the effect of the TAZ inhibitor, verteporfin (VP), on colon cancer HCT15 cells. Results of Western blotting showed that treatment with VP dose-dependently suppressed protein levels of TAZ and the pluripotent stem cell markers of Oct4 and Sox2 (Figure 5D). We also observed that treatment with VP down-regulated PODXL expression, suggesting that PODXL might also be regulated by TAZ (Figure 5D). Notably, treatment with 5-fluorouracil (5-FU) or irinotecan (CPT11) had less effect on HCT15 cells; however, the combination of VP with 5-FU or CPT11 significantly enhanced growth inhibition (Figure 5E) and tumorsphere formation (Figure 5F) compared to monotherapy. Together, these data indicate that PODXL plays an important role in drug resistance, and that developing therapeutic tools against PODXL is urgently needed for treating colon cancer.

## 3. Discussion

Although the clinical significance of PODXL expression in colon cancer has been reported, the underlying molecular mechanism is still unclear. Our present study shows the first time that the Hippo mechanism participates in downstream signaling of PODXL. Overexpression of PODXL associates with the YAP/TAZ core signature. Conversely, inhibition of PODXL decreases TAZ and TAZ downstream genes expression, and results in the suppression of tumor invasiveness, stemness, and sensitizing to chemotherapies. Moreover, coexpression of PODXL and TAZ renders the worst survival rate in colon cancer patients. Our findings demonstrate the potentiality of PODXL as a biomarker and therapeutic target for colon cancer.

Previous studies focused on the pro-invasive function of PODXL mainly through its ability in regulation of actin dynamics. The downstream genes regulated by PODXL are still unclear. Recently, Kusumoto et al., reported that PODXL regulates EMT genes in lung adenocarcinoma [19]. Expression of PODXL is responsible for transforming growth factor β-induced EMT gene production [20]. Additionally, PODXL also regulates tumor invasion by increasing expression of matrix metalloproteinase 9 [4,21]. Our study shows consistent results that expression of PODXL is associated with EMT genes in colon cancer patients, and, conversely, suppression of PODXL-downregulated EMT genes in colon cancer cells. Importantly, we found that the TAZ downstream genes and the stem cell-related genes were associated with PODXL. Knockdown of PODXL decreased TAZ and TAZ downstream genes expression.

TAZ is the key transducer of the Hippo signaling which plays a crucial role in organ homeostasis and has gained much attention on tumorigenesis over the past decade. Overexpression of TAZ has widely known to promote tumor initiation, metastasis, and stemness properties in various malignancies, and confers poor prognosis [22,23]. Additionally, previous studies show that expression of TAZ but not its orthologue YAP correlates to poor survival in colon cancer [24,25]. Our data confirmed that coexpression of PODXL with TAZ confers worst survival probability. Moreover, inhibition of PODXL as well as TAZ significantly decreased expression of stemness-related genes, and thus reduced self-renewal and drug-resistant capabilities. To the best of our knowledge, our study is the first to address the involvement of TAZ signaling in PODXL-mediated pro-tumor functions.

Though the core components of Hippo cascade are established, the upstream regulator, however, has not yet been fully understood. Recent studies reported that TAZ and YAP are the sensors of cellular structure and tension. High cell density lowers the level of F-actin and induces activation of Hippo cascade. Disruption of actin cytoskeleton blocks activation of YAP/TAZ [18]. On the contrary, stabilizing F-actin leads to dephosphorylate and nuclear accumulation of YAP [26]. Moreover, sphingosine-1-phosphate (S1P) and lysophosphatidic acid (LPA), ligands for G-protein-coupled receptors, have been shown to activate YAP by changes in F-actin levels [27]. These data support the notion that actin cytoskeleton regulates YAP/TAZ. It has shown that PODXL enhances motility and invasiveness by regulating actin cytoskeleton. PODXL increases actin-gelsolin interaction in cell protrusion [9]. We previously have reported that PODXL regulates actin polymerization through Rho GTPase Rac1/cdc42 signaling [11]. Rho GTPase family coordinates actin cytoskeleton, and its inhibition leads to the inactivation of YAP/TAZ [28]. Our present study showed that expression of PODXL associates with TAZ downstream gene expression. Suppression of PODXL induces phosphorylation of LATS1 and TAZ, and is accompanied with a decrease in TAZ protein expression. We speculate that changes in actin cytoskeleton may participate in PODXL-mediated TAZ signaling. Our findings uncover a novel upstream regulator of the Hippo mechanism.

## 4. Materials and Methods

### 4.1. Cell Culture and Chemicals

The human colon cancer cell lines of HCT116, LoVo, HT29, and HCT15 were obtained from the Bioresource Collection and Research Center (Hsinchu, Taiwan). Cells were cultured in RPMI medium containing 7% heat-inactivated fetal bovine serum (Gibco, Carlsbad, CA, USA), 100 units (U)/mL penicillin, and 100 U/mL streptomycin (Gibco). Cell lines were authenticated through a short-tandem repeat-profiling analysis to ensure that no culture contamination had occurred. Verteporfin (VP), 5-fluorouracil (5-FU), and irinotecan (CPT11) were purchased from Selleckchem (Houston, TX, USA) and were dissolved in dimethyl sulfoxide (Sigma, St. Louis, MO, USA). Antibodies against PODXL, TAZ, and phospho-TAZ were purchased from Santa Cruz Biotechnology (Santa Cruz, CA, USA). Antibodies against vimentin, Twist, Slug, and GADPH were obtained from GeneTex (San Antonio, TX, USA). Antibody against phospho-LATS1 was purchased from Cell Signaling Technology (Danvers, MA, USA)

### 4.2. Cell Viability and Colony Formation

Cell viability was determined by a 3-(4,5-dimethylthiazol-2-yl)-2,5-diphenyltetrazolium bromide (MTT) assay. Cells (5 × 10^4^) were seeded in a 24-well plate and treated with various concentrations of 5-FU or CPT11 for 48 h. Cells were then incubated with MTT (50 μg/mL) for 1 h. Formazan crystals were dissolved in isopropanol, and the absorbance at 570 nm was measured with a spectrophotometer. Cell viability is expressed as a percentage of the control group. The 50% inhibitory concentration (IC_50_) value was determined. For the colony formation assay, 10^4^ cells were seeded in a 6-well plate and treated with 5-FU or CPT11 for 7 days. Cells were then fixed, stained with a crystal violet solution, and observed under an inverted microscope.

### 4.3. Plasmid Transfection

HCT15 cells were seeded in a 6-well plate and transfected with pLKO-shRFP, pLKO-shPODXL (TRCN0000117019), or pLKO-shTAZ (TRCN0000019469) plasmids (RNAi core, Academia Sinica, Taipei, Taiwan) by PolyJET (SignaGen Laboratories, Ijamsville, MD, USA) for 48 h. Transfected cells were selected by puromycin (5 μg/mL) for 7 days and validated by real-time PCR assay [29].

### 4.4. Real-Time PCR

Total RNA was extracted with an RNA extraction kit and reverse-transcribed with a high-capacity cDNA conversion kit (Invitrogen, Carlsbad, CA, USA). cDNA was amplified using EvaGreen Master Mix (Biotium, Hayward, CA, USA) in the StepOne Plus Real-Time PCR system (Applied Biosystems, Darmstadt, Germany) with specific primers. Primer sequences are listed in Table S1. Results were calculated using the ΔΔ*C_T_* equation and are expressed as the multiple of change relative to a control sample.

### 4.5. Transwell Migration and Invasion

Cells at (1~4) × 10^5^ were seeded in a transwell insert (8-μm pore size, Corning Costar, New York, NY, USA) coated with or without Matrigel (BD Biosciences, Franklin Lakes, NJ, USA) for 24 (migration) and 48 h (invasion). Uninvaded cells were removed with a cotton swab, and cells which had passed through the lower membrane of the transwell were fixed, stained with a crystal violet solution, and observed under an inverted microscope.

### 4.6. Tumorsphere Formation Assay

Cells (10^3^) were seeded in a 6-well ultra-low attachment plate (Corning Costar) and maintained in serum-free DMEM-F12 supplemented with 20 ng/mL of epidermal growth factor (EGF; PeproTech, Rocky Hill, NJ, USA), 25 ng/mL basic fibroblast growth factor (bFGF; PeproTech), and B27 (Gibco) for 10 days. Formation of tumorspheres was observed and counted under a light microscope [30].

### 4.7. Western Blot Analysis

Cells were incubated with RIPA buffer (50 mM Tris-HCl (pH 7.4), 1% Nonidet P-40, 150 mM NaCl, 1 mM EGTA, and 0.025% sodium deoxycholate) supplemented with protease and phosphatase inhibitor cocktail (Roche Diagnostics Ltd, Mannheim, Germany) for 30 min on ice and centrifuged at 12,000 rpm for 30 min. Equal amounts of protein were separated by sodium dodecylsulfate (SDS)-polyacrylamide gel electrophoresis (PAGE) and then transferred onto polyvinylidene difluoride (PVDF) membranes. The membrane was blocked with 1% bovine serum albumin (BSA)/TBS-Tween 20 for 1 h and incubated overnight with the primary antibody. The membrane was washed with TBS-Tween 20 and further incubated with an appropriate horseradish peroxidase (HRP)-conjugated secondary antibody (GeneTex, San Antonio, TX, USA). Proteins were visualized using an enhanced chemiluminescence kit (Millipore, Temecula, CA, USA) and detected with a BioSpectrum Imaging system (UVP, Upland, CA, USA).

### 4.8. Database Analysis

Gene expression data of the PODXL, TAZ downstream, and EMT-associated genes were downloaded from the cancer genome atlas website (TCGA) for the colon and rectum adenocarcinoma project (COADREAD). Additionally, colorectal cancer mRNA-expressing datasets of GSE17536 [31], GSE41258 [32], and GSE68468 [32] were downloaded from the gene expression omnibus (GEO) website for data mining. A correlation coefficient was determined by Spearman’s test. A survival curve was created by the Kaplan-Meier analysis. Statistical differences were based on the log-rank test.

### 4.9. Statistical Analyses

Each experiment was performed in triplicate, and results are expressed as the mean ± standard error (SE). The significance of the difference from the respective controls for each experimental test condition was assayed using a two-tailed Student’s *t*-test. * *p* < 0.05 or ** *p* < 0.01, *** *p* < 0.001 was regarded as a significant difference related to the indicated group. Statistical analyses were carried out with GraphPad Prism 5 software.

## 5. Conclusions

We demonstrated a novel mechanism underlying PODXL-mediated invasiveness and cancer stemness through regulating TAZ signaling. Our findings can be used to develop powerful diagnostic or therapeutic tools for treating colon cancer.

## Supplementary Materials

Supplementary materials can be found at www.mdpi.com/1422-0067/18/10/2047/s1.

## References

[1] Armaghany T., Wilson J. D., Chu Q., Mills G. (2012). Genetic alterations in colorectal cancer. Gastrointest. Cancer Res..

[2] Colussi D., Brandi G., Bazzoli F., Ricciardiello L. (2013). Molecular pathways involved in colorectal cancer: Implications for disease behavior and prevention. Int. J. Mol. Sci..

[3] Meder D., Shevchenko A., Simons K., Fullekrug J. (2005). Gp135/podocalyxin and NHERF-2 participate in the formation of a preapical domain during polarization of MDCK cells. J. Cell Biol..

[4] Sizemore S., Cicek M., Sizemore N., Ng K.P., Casey G. (2007). Podocalyxin increases the aggressive phenotype of breast and prostate cancer cells in vitro through its interaction with ezrin. Cancer Res..

[5] Larrucea S., Butta N., Arias-Salgado E.G., Alonso-Martin S., Ayuso M.S., Parrilla R. (2008). Expression of podocalyxin enhances the adherence, migration, and intercellular communication of cells. Exp. Cell Res..

[6] Takeda T., McQuistan T., Orlando R.A., Farquhar M.G. (2001). Loss of glomerular foot processes is associated with uncoupling of podocalyxin from the actin cytoskeleton. J. Clin. Investig..

[7] Forse C. L., Yilmaz Y.E., Pinnaduwage D., O’Malley F.P., Mulligan A.M., Bull S.B., Andrulis I.L. (2013). Elevated expression of podocalyxin is associated with lymphatic invasion, basal-like phenotype, and clinical outcome in axillary lymph node-negative breast cancer. Breast Cancer Res. Treat..

[8] Binder Z.A., Siu I.M., Eberhart C.G., Ap Rhys C., Bai R.Y., Staedtke V., Zhang H., Smoll N.R., Piantadosi S., Piccirillo S.G. (2013). Podocalyxin-like protein is expressed in glioblastoma multiforme stem-like cells and is associated with poor outcome. PLoS ONE.

[9] Taniuchi K., Furihata M., Naganuma S., Dabanaka K., Hanazaki K., Saibara T. (2016). Podocalyxin-like protein, linked to poor prognosis of pancreatic cancers, promotes cell invasion by binding to gelsolin. Cancer Sci..

[10] Kaprio T., Fermer C., Hagstrom J., Mustonen H., Bockelman C., Nilsson O., Haglund C. (2014). Podocalyxin is a marker of poor prognosis in colorectal cancer. BMC Cancer.

[11] Lin C. W., Sun M. S., Liao M. Y., Chung C. H., Chi Y. H., Chiou L. T., Yu J., Lou K. L., Wu H. C. (2014). Podocalyxin-like 1 promotes invadopodia formation and metastasis through activation of Rac1/Cdc42/cortactin signaling in breast cancer cells. Carcinogenesis.

[12] Hsu Y.H., Lin W.L., Hou Y.T., Pu Y.S., Shun C.T., Chen C.L., Wu Y.Y., Chen J.Y., Chen T.H., Jou T.S. (2010). Podocalyxin EBP50 ezrin molecular complex enhances the metastatic potential of renal cell carcinoma through recruiting Rac1 guanine nucleotide exchange factor ARHGEF7. Am. J. Pathol..

[13] Mo J.S., Park H.W., Guan K.L. (2014). The Hippo signaling pathway in stem cell biology and cancer. EMBO Rep..

[14] Zhang K., Qi H.X., Hu Z.M., Chang Y.N., Shi Z.M., Han X.H., Han Y.W., Zhang R.X., Zhang Z., Chen T. (2015). YAP and TAZ take center stage in cancer. Biochemistry.

[15] Piccolo S., Cordenonsi M., Dupont S. (2013). Molecular pathways: YAP and TAZ take center stage in organ growth and tumorigenesis. Clin. Cancer Res..

[16] Halder G., Dupont S., Piccolo S. (2012). Transduction of mechanical and cytoskeletal cues by YAP and TAZ. Nat. Rev. Mol. Cell Biol..

[17] Dupont S. (2016). Role of YAP/TAZ in cell-matrix adhesion-mediated signalling and mechanotransduction. Exp. Cell Res..

[18] Mana-Capelli S., Paramasivam M., Dutta S., McCollum D. (2014). Angiomotins link F-actin architecture to Hippo pathway signaling. Mol. Biol. Cell.

[19] Kusumoto H., Shintani Y., Kanzaki R., Kawamura T., Funaki S., Minami M., Nagatomo I., Morii E., Okumura M. (2017). Podocalyxin influences malignant potential by controlling epithelial-mesenchymal transition in lung adenocarcinoma. Cancer Sci..

[20] Meng X., Ezzati P., Wilkins J.A. (2011). Requirement of podocalyxin in TGF-beta induced epithelial mesenchymal transition. PLoS ONE.

[21] Liu Y., Yang L., Liu B., Jiang Y.G. (2014). Podocalyxin promotes glioblastoma multiforme cell invasion and proliferation via beta-catenin signaling. PLoS ONE.

[22] Li Z., Wang Y., Zhu Y., Yuan C., Wang D., Zhang W., Qi B., Qiu J., Song X., Ye J. (2015). The Hippo transducer TAZ promotes epithelial to mesenchymal transition and cancer stem cell maintenance in oral cancer. Mol. Oncol..

[23] Bartucci M., Dattilo R., Moriconi C., Pagliuca A., Mottolese M., Federici G., Benedetto A.D., Todaro M., Stassi G., Sperati F. (2015). TAZ is required for metastatic activity and chemoresistance of breast cancer stem cells. Oncogene.

[24] Wang L., Shi S., Guo Z., Zhang X., Han S., Yang A., Wen W., Zhu Q. (2013). Overexpression of YAP and TAZ is an independent predictor of prognosis in colorectal cancer and related to the proliferation and metastasis of colon cancer cells. PLoS ONE.

[25] Yuen H.F., McCrudden C.M., Huang Y.H., Tham J.M., Zhang X., Zeng Q., Zhang S.D., Hong W. (2013). TAZ expression as a prognostic indicator in colorectal cancer. PLoS ONE.

[26] Zhao B., Li L., Wang L., Wang C.Y., Yu J., Guan K.L. (2012). Cell detachment activates the Hippo pathway via cytoskeleton reorganization to induce anoikis. Genes Dev..

[27] Miller E., Yang J., DeRan M., Wu C., Su A.I., Bonamy G.M., Liu J., Peters E.C., Wu X. (2012). Identification of serum-derived sphingosine-1-phosphate as a small molecule regulator of YAP. Chem. Biol..

[28] Yu F.X., Zhao B., Panupinthu N., Jewell J.L., Lian I., Wang L.H., Zhao J., Yuan H., Tumaneng K., Li H. (2012). Regulation of the Hippo-YAP pathway by G-protein-coupled receptor signaling. Cell.

[29] Lee W.Y., Lee W.T., Cheng C.H., Chen K.C., Chou C.M., Chung C.H., Sun M.S., Cheng H.W., Ho M.N., Lin C.W. (2015). Repositioning antipsychotic chlorpromazine for treating colorectal cancer by inhibiting sirtuin 1. Oncotarget.

[30] Lin C.W., Liao M.Y., Lin W.W., Wang Y.P., Lu T.Y., Wu H.C. (2012). Epithelial cell adhesion molecule regulates tumor initiation and tumorigenesis via activating reprogramming factors and epithelial-mesenchymal transition gene expression in colon cancer. J. Biol. Chem..

[31] Smith J.J., Deane N.G., Wu F., Merchant N.B., Zhang B., Jiang A., Lu P., Johnson J.C., Schmidt C., Bailey C.E. (2010). Experimentally derived metastasis gene expression profile predicts recurrence and death in patients with colon cancer. Gastroenterology.

[32] Sheffer M., Bacolod M.D., Zuk O., Giardina S.F., Pincas H., Barany F., Paty P.B., Gerald W.L., Notterman D.A., Domany E. (2009). Association of survival and disease progression with chromosomal instability: A genomic exploration of colorectal cancer. Proc. Natl. Acad. Sci. USA.

