# Supplementary material for: Podocalyxin-Like Protein 1 Regulates TAZ Signaling and Stemness Properties in Colon Cancer
Source: Int J Mol Sci. 2017 Sep 23;18(10):2047. doi: 10.3390/ijms18102047 (PMC5666729; doi:10.3390/ijms18102047)
Supplement: Supplementary file 1 [file ijms-18-02047-s001.pdf]

Table S1. List of oligonucleotides for the real-time PCR assay.

Gene	Forward	Reverse
Amphiregulin	GGGAGTGAGATTTCCCCTGT	AGCCAGGTATTTGTGGTTCG
ABCG2	CACCTTATTGGCCTCAGGAA	CCTGCTTGGAAGGCTCTAGT
ALDH	CCGTGGCGTACTATGGATGC	GCAGCAGACGATCTCTTTCGAT
AXL	TTTCCTGAGTGA AGCGGTCT	CATCTGAGTGGGCAGGTACA
Survivin	GGCCCAGTGTTTCTTCTGCTT	GCAACCGGACGAATGCTTT
CYR61	GAGTGGGTCTGTGACGAGGAT	GGTTGTATAGGATGCGAGGCT
CTGF	TGACCTGGAGGAAAACATTAAGA	AGCCCTGTATGTCTTCACACTG
CyclinD1	AAGTGCGTGCAGAAGGAGATTGTG	TCGGGCCGGATAGAGTTGTCAGT
E-cadherin	GGAACTATGAAAAGTGGGCTTG	AAATTGCCAGGCTCAATGAC
EpCAM	CTGGCCGTAAACTGCTTTGT	AGCCCATCATTGTTCTGGAG
Fibronectin	GAACTATGATGCCGACCAGAA	GGTTGTGCAGATTTCCTCGT
GAPDH	CTTCACCACCATGGAGGAGGC	GGCATGGACTGTGGTCATGAG
Nanog	GTCCCGGTCAAGAAACAGAA	TGCGTCACACCATTGCTATT
Oct4	ATTCAGCCAAACGACCATCT	ACACTCGGACCACATCCTTC
PODXL	AAGGCCAGGGGTTCACAT	AGCCTCGCATCCCTCTAACT
Sox2	ATGGGTTCGGTGGTCAAGT	ATGTGTGAGAGGGGCAGTGT
TAZ	CAGCCAAATCTCGTGATGAATC	GGTTCTGCTGGCTCAGGGT
Twist	GGCATCACTATGGACTTTCTCTATT	GGCCAGTTTGATCCCAGTATT
Vimentin	TGTCCAAATCGATGTGGATGTTTC	TTGTACCATTCTTCTGCCTCCTG
Zeb1	GGGAGGAGCAGTGAAAGAGA	TTTCTTGCCCTTCCTTTCTG
